# (*E*)-1-{4-[(*E*)-3-Chloro­benzyl­idene­amino]­phen­yl}-3-(3-chloro­phen­yl)prop-2-en-1-one

**DOI:** 10.1107/S1600536811013778

**Published:** 2011-04-29

**Authors:** Jian-Ming Cheng, Yun-Feng Zheng, Guo-Ping Peng

**Affiliations:** a College of Pharmacy, Nanjing University of Chinese Medicine, Nanjing 210029, People’s Republic of China

## Abstract

In the title mol­ecule, C_22_H_15_Cl_2_NO, the dihedral angles between the central aromatic ring and the N- and C=O-bonded rings are 43.13 (13) and 0.80 (14)°, respectively. The dihedral angle between the terminal rings is 43.15 (14)°. The major twist occurs about the C_ar_—N bond [C_ar_—C_ar_—N=C = 42.3 (4)°; ar is aromatic].

## Related literature

For background to Schiff bases, see: Chimenti *et al.* (2009 ▶); Shi *et al.* (2007 ▶). For reference bond lengths, see: Allen *et al.* (1987 ▶).
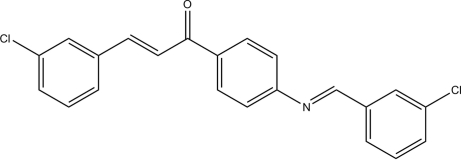


## Experimental

### 

#### Crystal data

C_22_H_15_Cl_2_NO
*M* *_r_* = 380.25
Monoclinic, 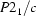

*a* = 17.454 (4) Å
*b* = 6.1110 (12) Å
*c* = 17.179 (3) Å
β = 100.32 (3)°
*V* = 1802.7 (6) Å^3^
*Z* = 4
Mo *K*α radiation
μ = 0.37 mm^−1^
*T* = 293 K
0.40 × 0.30 × 0.10 mm

#### Data collection

Enraf–Nonius CAD-4 diffractometer
Absorption correction: ψ scan (North *et al.*, 1968 ▶) *T*_min_ = 0.866, *T*_max_ = 0.964
3659 measured reflections
3539 independent reflections
2367 reflections with *I* > 2σ(*I*)
*R*_int_ = 0.021
200 standard reflections every 3 reflections intensity decay: 1%

#### Refinement

*R*[*F* ^2^ > 2σ(*F* ^2^)] = 0.057
*wR*(*F* ^2^) = 0.145
*S* = 1.06
3539 reflections
235 parameters
H-atom parameters constrained
Δρ_max_ = 0.26 e Å^−3^
Δρ_min_ = −0.37 e Å^−3^

### 

Data collection: *CAD-4 Software* (Enraf–Nonius, 1989 ▶); cell refinement: *CAD-4 Software*; data reduction: *XCAD4* (Harms & Wocadlo, 1995 ▶); program(s) used to solve structure: *SHELXS97* (Sheldrick, 2008 ▶); program(s) used to refine structure: *SHELXL97* (Sheldrick, 2008 ▶); molecular graphics: *SHELXTL* (Sheldrick, 2008 ▶); software used to prepare material for publication: *SHELXTL*.

## Supplementary Material

Crystal structure: contains datablocks global, I. DOI: 10.1107/S1600536811013778/hb5842sup1.cif

Structure factors: contains datablocks I. DOI: 10.1107/S1600536811013778/hb5842Isup2.hkl

Additional supplementary materials:  crystallographic information; 3D view; checkCIF report

## Figures and Tables

**Fig. 1.**
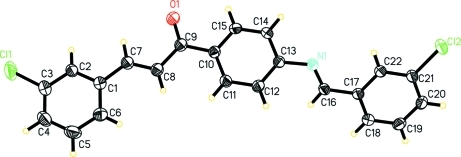
The structure of (I) showing 30% probability displacement ellipsoids.

## supplementary crystallographic information

### Comment

There has been much research interest in Schiff base and chalcone compounds due to their biological activities (Shi *et al.*, 2007; Chimenti *et al.*, 2009). In this work, we report here the crystal structure of the title compound, (I). In (I), all bond lengths are within normal ranges (Allen *et al.*, 1987) (Fig. 1).

### Experimental

The title compound was prepared by stirring a mixture of 3-chlorobenzaldehyde (280 mg, 2 mmol) and 1-(4-aminophenyl)ethanone (135 mg, 1 mmol) in methanol (10 ml) for 4 h. After keeping the filtrate in air for 5 d, colorless block-shaped crystals of (I) were formed.

### Refinement

All H atoms were positioned geometrically (C—H = 0.93 Å for the aromatic H atoms and C—H = 0.96 Å for the aliphatic H atoms) and were refined as riding, with *U*_iso_(H) = 1.2*U*_eq_(C) and *U*_iso_(H) = 1.2*U*_eq_(N).

### Crystal data

**Table:**

C_22_H_15_Cl_2_NO	*F*(000) = 784
*M**_r_* = 380.25	*D*_x_ = 1.401 Mg m^−^^3^
Monoclinic, *P*2_1_/*c*	Mo *K*α radiation, λ = 0.71073 Å
Hall symbol: -P 2ybc	Cell parameters from 25 reflections
*a* = 17.454 (4) Å	θ = 9–12°
*b* = 6.1110 (12) Å	µ = 0.37 mm^−^^1^
*c* = 17.179 (3) Å	*T* = 293 K
β = 100.32 (3)°	Block, colorless
*V* = 1802.7 (6) Å^3^	0.40 × 0.30 × 0.10 mm
*Z* = 4	

### Data collection

**Table:**

Enraf–Nonius CAD-4 diffractometer	2367 reflections with *I* > 2σ(*I*)
Radiation source: fine-focus sealed tube	*R*_int_ = 0.021
graphite	θ_max_ = 26.0°, θ_min_ = 1.2°
ω/2θ scans	*h* = −21→0
Absorption correction: ψ scan (North *et al.*, 1968)	*k* = 0→7
*T*_min_ = 0.866, *T*_max_ = 0.964	*l* = −20→21
3659 measured reflections	200 standard reflections every 3 reflections
3539 independent reflections	intensity decay: 1%

### Refinement

**Table:**

Refinement on *F*^2^	Primary atom site location: structure-invariant direct methods
Least-squares matrix: full	Secondary atom site location: difference Fourier map
*R*[*F*^2^ > 2σ(*F*^2^)] = 0.057	Hydrogen site location: inferred from neighbouring sites
*wR*(*F*^2^) = 0.145	H-atom parameters constrained
*S* = 1.06	*w* = 1/[σ^2^(*F*_o_^2^) + (0.0633*P*)^2^ + 0.6746*P*] where *P* = (*F*_o_^2^ + 2*F*_c_^2^)/3
3539 reflections	(Δ/σ)_max_ < 0.001
235 parameters	Δρ_max_ = 0.26 e Å^−^^3^
0 restraints	Δρ_min_ = −0.37 e Å^−^^3^

### Special details

**Table:**

Geometry. All e.s.d.'s (except the e.s.d. in the dihedral angle between two l.s. planes) are estimated using the full covariance matrix. The cell e.s.d.'s are taken into account individually in the estimation of e.s.d.'s in distances, angles and torsion angles; correlations between e.s.d.'s in cell parameters are only used when they are defined by crystal symmetry. An approximate (isotropic) treatment of cell e.s.d.'s is used for estimating e.s.d.'s involving l.s. planes.
Refinement. Refinement of *F*^2^ against ALL reflections. The weighted *R*-factor *wR* and goodness of fit *S* are based on *F*^2^, conventional *R*-factors *R* are based on *F*, with *F* set to zero for negative *F*^2^. The threshold expression of *F*^2^ > σ(*F*^2^) is used only for calculating *R*-factors(gt) *etc*. and is not relevant to the choice of reflections for refinement. *R*-factors based on *F*^2^ are statistically about twice as large as those based on *F*, and *R*- factors based on ALL data will be even larger.

### Fractional atomic coordinates and isotropic or equivalent isotropic displacement parameters (Å^2^)

**Table:**

	*x*	*y*	*z*	*U*_iso_*/*U*_eq_	
C1	0.82827 (15)	−0.0032 (5)	−0.05196 (15)	0.0486 (7)	
C2	0.87296 (15)	−0.1135 (5)	−0.09848 (16)	0.0524 (7)	
H2	0.8585	−0.2535	−0.1167	0.063*	
C3	0.93831 (17)	−0.0187 (6)	−0.11798 (18)	0.0617 (8)	
C4	0.96089 (19)	0.1871 (6)	−0.0928 (2)	0.0732 (9)	
H4	1.0054	0.2501	−0.1058	0.088*	
C5	0.9159 (2)	0.2994 (6)	−0.0476 (2)	0.0802 (10)	
H5	0.9301	0.4405	−0.0307	0.096*	
C6	0.85060 (18)	0.2070 (5)	−0.02714 (19)	0.0652 (8)	
H6	0.8213	0.2855	0.0035	0.078*	
C7	0.76114 (15)	−0.1165 (5)	−0.03071 (15)	0.0515 (7)	
H7	0.7557	−0.2622	−0.0462	0.062*	
C8	0.70704 (15)	−0.0416 (5)	0.00740 (15)	0.0515 (7)	
H8	0.7078	0.1040	0.0232	0.062*	
C9	0.64549 (16)	−0.1899 (5)	0.02472 (15)	0.0498 (7)	
C10	0.58642 (15)	−0.1109 (4)	0.07094 (14)	0.0428 (6)	
C11	0.58854 (15)	0.0934 (4)	0.10662 (15)	0.0488 (7)	
H11	0.6285	0.1902	0.1017	0.059*	
C12	0.53180 (15)	0.1548 (5)	0.14945 (15)	0.0475 (6)	
H12	0.5344	0.2910	0.1740	0.057*	
C13	0.47135 (15)	0.0137 (4)	0.15568 (14)	0.0429 (6)	
C14	0.46980 (17)	−0.1910 (4)	0.12070 (17)	0.0551 (7)	
H14	0.4299	−0.2884	0.1254	0.066*	
C15	0.52654 (16)	−0.2513 (5)	0.07917 (16)	0.0525 (7)	
H15	0.5246	−0.3893	0.0561	0.063*	
C16	0.37984 (14)	0.2508 (4)	0.18899 (14)	0.0441 (6)	
H16	0.4007	0.3531	0.1586	0.053*	
C17	0.31366 (15)	0.3162 (4)	0.22540 (14)	0.0435 (6)	
C18	0.28087 (16)	0.5218 (5)	0.20967 (16)	0.0515 (7)	
H18	0.3023	0.6187	0.1777	0.062*	
C19	0.21688 (17)	0.5833 (5)	0.24099 (18)	0.0591 (8)	
H19	0.1958	0.7223	0.2307	0.071*	
C20	0.18380 (17)	0.4406 (5)	0.28750 (18)	0.0598 (8)	
H20	0.1405	0.4819	0.3087	0.072*	
C21	0.21605 (16)	0.2352 (5)	0.30206 (16)	0.0522 (7)	
C22	0.28056 (15)	0.1702 (4)	0.27221 (14)	0.0456 (6)	
H22	0.3017	0.0315	0.2831	0.055*	
Cl1	0.99327 (5)	−0.1679 (2)	−0.17471 (6)	0.0968 (4)	
Cl2	0.17205 (5)	0.05098 (15)	0.35786 (5)	0.0771 (3)	
N1	0.40982 (12)	0.0634 (4)	0.19679 (12)	0.0468 (5)	
O1	0.64304 (12)	−0.3797 (4)	0.00131 (13)	0.0693 (6)	

### Atomic displacement parameters (Å^2^)

**Table:**

	*U*^11^	*U*^22^	*U*^33^	*U*^12^	*U*^13^	*U*^23^
C1	0.0474 (15)	0.0542 (17)	0.0434 (14)	−0.0010 (13)	0.0060 (12)	−0.0006 (13)
C2	0.0494 (15)	0.0540 (17)	0.0555 (16)	−0.0054 (13)	0.0142 (13)	−0.0054 (14)
C3	0.0508 (17)	0.073 (2)	0.0626 (18)	−0.0045 (16)	0.0147 (14)	0.0011 (16)
C4	0.0557 (18)	0.076 (2)	0.088 (2)	−0.0150 (18)	0.0127 (17)	0.004 (2)
C5	0.080 (2)	0.056 (2)	0.101 (3)	−0.0187 (19)	0.008 (2)	−0.006 (2)
C6	0.0644 (19)	0.058 (2)	0.074 (2)	−0.0058 (16)	0.0129 (16)	−0.0152 (16)
C7	0.0545 (16)	0.0551 (17)	0.0463 (15)	−0.0052 (14)	0.0128 (13)	−0.0080 (13)
C8	0.0561 (16)	0.0539 (17)	0.0465 (15)	−0.0015 (14)	0.0147 (13)	−0.0061 (13)
C9	0.0544 (16)	0.0524 (18)	0.0433 (14)	−0.0001 (14)	0.0105 (12)	−0.0051 (13)
C10	0.0476 (14)	0.0451 (15)	0.0367 (13)	0.0012 (12)	0.0101 (11)	−0.0005 (11)
C11	0.0463 (15)	0.0496 (17)	0.0521 (15)	−0.0084 (13)	0.0134 (12)	−0.0080 (13)
C12	0.0504 (15)	0.0467 (15)	0.0462 (15)	−0.0026 (13)	0.0112 (12)	−0.0085 (13)
C13	0.0509 (15)	0.0417 (15)	0.0379 (13)	0.0044 (12)	0.0127 (11)	0.0077 (12)
C14	0.0620 (17)	0.0414 (16)	0.0688 (18)	−0.0072 (14)	0.0305 (15)	0.0025 (14)
C15	0.0658 (17)	0.0367 (15)	0.0588 (17)	−0.0037 (13)	0.0216 (14)	−0.0044 (13)
C16	0.0491 (14)	0.0444 (15)	0.0410 (14)	−0.0059 (13)	0.0145 (11)	0.0018 (12)
C17	0.0463 (14)	0.0418 (15)	0.0432 (13)	−0.0053 (12)	0.0102 (11)	−0.0051 (12)
C18	0.0541 (16)	0.0446 (16)	0.0576 (17)	−0.0030 (13)	0.0151 (13)	−0.0005 (13)
C19	0.0590 (17)	0.0460 (17)	0.074 (2)	0.0038 (14)	0.0155 (15)	−0.0092 (15)
C20	0.0563 (17)	0.059 (2)	0.070 (2)	−0.0035 (15)	0.0259 (15)	−0.0166 (16)
C21	0.0573 (17)	0.0535 (18)	0.0505 (16)	−0.0132 (14)	0.0218 (13)	−0.0090 (13)
C22	0.0502 (15)	0.0432 (15)	0.0455 (14)	−0.0051 (12)	0.0141 (12)	−0.0026 (12)
Cl1	0.0694 (6)	0.1239 (9)	0.1101 (8)	−0.0134 (6)	0.0507 (5)	−0.0227 (7)
Cl2	0.0919 (6)	0.0759 (6)	0.0763 (5)	−0.0213 (5)	0.0496 (5)	−0.0045 (4)
N1	0.0545 (13)	0.0444 (13)	0.0452 (12)	0.0037 (11)	0.0192 (10)	0.0070 (10)
O1	0.0787 (15)	0.0571 (13)	0.0810 (15)	−0.0079 (11)	0.0379 (12)	−0.0226 (11)

### Geometric parameters (Å, °)

**Table:**

C1—C6	1.387 (4)	C12—C13	1.382 (4)
C1—C2	1.387 (4)	C12—H12	0.9300
C1—C7	1.462 (4)	C13—C14	1.386 (4)
C2—C3	1.373 (4)	C13—N1	1.420 (3)
C2—H2	0.9300	C14—C15	1.370 (4)
C3—C4	1.365 (5)	C14—H14	0.9300
C3—Cl1	1.742 (3)	C15—H15	0.9300
C4—C5	1.382 (5)	C16—N1	1.256 (3)
C4—H4	0.9300	C16—C17	1.464 (3)
C5—C6	1.373 (4)	C16—H16	0.9300
C5—H5	0.9300	C17—C18	1.387 (4)
C6—H6	0.9300	C17—C22	1.394 (3)
C7—C8	1.323 (4)	C18—C19	1.376 (4)
C7—H7	0.9300	C18—H18	0.9300
C8—C9	1.476 (4)	C19—C20	1.377 (4)
C8—H8	0.9300	C19—H19	0.9300
C9—O1	1.225 (3)	C20—C21	1.380 (4)
C9—C10	1.490 (3)	C20—H20	0.9300
C10—C15	1.379 (4)	C21—C22	1.377 (4)
C10—C11	1.388 (4)	C21—Cl2	1.743 (3)
C11—C12	1.387 (3)	C22—H22	0.9300
C11—H11	0.9300		
			
C6—C1—C2	118.1 (3)	C13—C12—H12	120.0
C6—C1—C7	123.7 (3)	C11—C12—H12	120.0
C2—C1—C7	118.2 (3)	C12—C13—C14	119.1 (2)
C3—C2—C1	120.8 (3)	C12—C13—N1	124.1 (2)
C3—C2—H2	119.6	C14—C13—N1	116.8 (2)
C1—C2—H2	119.6	C15—C14—C13	120.5 (3)
C4—C3—C2	121.1 (3)	C15—C14—H14	119.7
C4—C3—Cl1	120.2 (2)	C13—C14—H14	119.7
C2—C3—Cl1	118.7 (3)	C14—C15—C10	121.2 (3)
C3—C4—C5	118.3 (3)	C14—C15—H15	119.4
C3—C4—H4	120.8	C10—C15—H15	119.4
C5—C4—H4	120.8	N1—C16—C17	123.3 (2)
C6—C5—C4	121.4 (3)	N1—C16—H16	118.4
C6—C5—H5	119.3	C17—C16—H16	118.4
C4—C5—H5	119.3	C18—C17—C22	119.5 (2)
C5—C6—C1	120.2 (3)	C18—C17—C16	119.6 (2)
C5—C6—H6	119.9	C22—C17—C16	120.8 (2)
C1—C6—H6	119.9	C19—C18—C17	120.5 (3)
C8—C7—C1	129.4 (3)	C19—C18—H18	119.8
C8—C7—H7	115.3	C17—C18—H18	119.8
C1—C7—H7	115.3	C18—C19—C20	120.5 (3)
C7—C8—C9	119.8 (3)	C18—C19—H19	119.8
C7—C8—H8	120.1	C20—C19—H19	119.8
C9—C8—H8	120.1	C19—C20—C21	118.8 (3)
O1—C9—C8	119.9 (2)	C19—C20—H20	120.6
O1—C9—C10	119.7 (3)	C21—C20—H20	120.6
C8—C9—C10	120.4 (2)	C22—C21—C20	121.9 (3)
C15—C10—C11	118.3 (2)	C22—C21—Cl2	119.3 (2)
C15—C10—C9	117.6 (2)	C20—C21—Cl2	118.8 (2)
C11—C10—C9	124.1 (2)	C21—C22—C17	118.8 (3)
C12—C11—C10	120.8 (2)	C21—C22—H22	120.6
C12—C11—H11	119.6	C17—C22—H22	120.6
C10—C11—H11	119.6	C16—N1—C13	118.7 (2)
C13—C12—C11	120.0 (2)		
			
C6—C1—C2—C3	1.3 (4)	C11—C12—C13—C14	1.9 (4)
C7—C1—C2—C3	−177.6 (3)	C11—C12—C13—N1	−178.7 (2)
C1—C2—C3—C4	−0.6 (5)	C12—C13—C14—C15	−1.3 (4)
C1—C2—C3—Cl1	178.6 (2)	N1—C13—C14—C15	179.3 (3)
C2—C3—C4—C5	−0.6 (5)	C13—C14—C15—C10	−0.1 (4)
Cl1—C3—C4—C5	−179.7 (3)	C11—C10—C15—C14	0.7 (4)
C3—C4—C5—C6	0.9 (6)	C9—C10—C15—C14	−179.9 (3)
C4—C5—C6—C1	−0.2 (5)	N1—C16—C17—C18	−175.9 (3)
C2—C1—C6—C5	−0.9 (4)	N1—C16—C17—C22	0.6 (4)
C7—C1—C6—C5	177.9 (3)	C22—C17—C18—C19	1.1 (4)
C6—C1—C7—C8	6.8 (5)	C16—C17—C18—C19	177.6 (3)
C2—C1—C7—C8	−174.4 (3)	C17—C18—C19—C20	−0.9 (4)
C1—C7—C8—C9	−177.9 (3)	C18—C19—C20—C21	0.0 (4)
C7—C8—C9—O1	−2.6 (4)	C19—C20—C21—C22	0.7 (4)
C7—C8—C9—C10	177.1 (2)	C19—C20—C21—Cl2	−177.7 (2)
O1—C9—C10—C15	−5.9 (4)	C20—C21—C22—C17	−0.5 (4)
C8—C9—C10—C15	174.4 (2)	Cl2—C21—C22—C17	177.81 (19)
O1—C9—C10—C11	173.4 (3)	C18—C17—C22—C21	−0.4 (4)
C8—C9—C10—C11	−6.2 (4)	C16—C17—C22—C21	−176.9 (2)
C15—C10—C11—C12	0.0 (4)	C17—C16—N1—C13	176.9 (2)
C9—C10—C11—C12	−179.4 (2)	C12—C13—N1—C16	42.3 (4)
C10—C11—C12—C13	−1.3 (4)	C14—C13—N1—C16	−138.3 (3)
